# Evaluation of Chromosome Microarray Analysis in a Large Cohort of Females with Autism Spectrum Disorders: A Single Center Italian Study

**DOI:** 10.3390/jpm10040160

**Published:** 2020-10-09

**Authors:** Sara Calderoni, Ivana Ricca, Giulia Balboni, Romina Cagiano, Denise Cassandrini, Stefano Doccini, Angela Cosenza, Deborah Tolomeo, Raffaella Tancredi, Filippo Maria Santorelli, Filippo Muratori

**Affiliations:** 1 Department of Developmental Neuroscience, IRCCS Fondazione Stella Maris, Viale del Tirreno 331, Calambrone, 56128 Pisa, Italy; romina.cagiano@fsm.unipi.it (R.C.); angela.cosenza@fsm.unipi.it (A.C.); r.tancredi@fsm.unipi.it (R.T.); f.muratori@fsm.unipi.it (F.M.); 2 Department of Clinical and Experimental Medicine, University of Pisa, Via Savi, 10, 56126 Pisa, Italy; 3 Molecular Medicine, IRCCS Fondazione Stella Maris, via dei Giacinti 2, Calambrone, 56128 Pisa, Italy; ivana.ricca@fsm.unipi.it (I.R.); d.cassandrini@fsm.unipi.it (D.C.); s.doccini@fsm.unipi.it (S.D.); d.tolomeo@fsm.unipi.it (D.T.); f.santorelli@fsm.unipi.it (F.M.S.); 4 Department of Philosophy, Social and Human Sciences and Education, University of Perugia, Piazza G. Ermini 1, 06123 Perugia, Italy; giulia.balboni@unipg.it; 5 Department of Neurosciences, Psychology, Drug Research and Child Health (NEUROFARBA), University of Florence, Viale Pieraccini, 6-50139 Florence, Italy

**Keywords:** autism spectrum disorders, copy number variants, females, Array-Comparative Genomic Hybridization (Array-CGH)

## Abstract

Autism spectrum disorders (ASD) encompass a heterogeneous group of neurodevelopmental disorders resulting from the complex interaction between genetic and environmental factors. Thanks to the chromosome microarray analysis (CMA) in clinical practice, the accurate identification and characterization of submicroscopic deletions/duplications (copy number variants, CNVs) associated with ASD was made possible. However, the widely acknowledged excess of males on the autism spectrum reflects on a paucity of CMA studies specifically focused on females with ASD (f-ASD). In this framework, we aim to evaluate the frequency of causative CNVs in a single-center cohort of idiopathic f-ASD. Among the 90 f-ASD analyzed, we found 20 patients with one or two potentially pathogenic CNVs, including those previously associated with ASD (located at 16*p*13.2 16*p*11.2, 15*q*11.2, and 22*q*11.21 regions). An exploratory genotype/phenotype analysis revealed that the f-ASD with causative CNVs had statistically significantly lower restrictive and repetitive behaviors than those without CNVs or with non-causative CNVs. Future work should focus on further understanding of f-ASD genetic underpinnings, taking advantage of next-generation sequencing technologies, with the ultimate goal of contributing to precision medicine in ASD.

## 1. Introduction

Autism spectrum disorders (ASD) are a heterogeneous group of neurodevelopmental pathologies characterized by early onset abnormalities in social communication and interaction, as well as atypically restricted and repetitive behaviors and interests [1]. Despite the exact pathogenesis of idiopathic ASD not yet being fully elucidated, recent evidences suggest an interaction between genetic liability and environmental influences in producing early alteration of brain development [2]. In particular, among environmental risk factors, several maternal factors (including age ≥ 35 years, chronic hypertension, preeclampsia, gestational hypertension, and overweight before or during pregnancy) were associated with ASD in an updated review of the literature [3]. Updated data on the prevalence of ASD in the US (Centers for Disease Control and Prevention, CDC [4]) identified 1 in 54 children as having ASD, while the estimated prevalence of ASD in Italian population is 1 in 87, according to a recent investigation [5].

Crucially, since the first descriptions of autism [6,7], a strong male bias in ASD prevalence has been consistently observed, which becomes even more pronounced in individuals without intellectual disability, according to data from the 80s [8,9]. More recent studies have challenged this assertion, suggesting that missed or wrong diagnoses of ASD females, especially of those with good intellectual and language abilities, contribute to the skewed sex ratio in ASD [10].

The exact mechanisms underlying male vulnerability or female protection in ASD remain complex and scarcely investigated. A multifactorial model has been proposed where a mixture of gene variants and environmental factors contribute to liability, possibly interacting with sex-specific pathways such as those related to hormones or immune function [11,12].

Genetic investigations in ASD revealed frequently sexually dimorphic results. For example, a greater number of de novo copy number variants (CNVs) [13,14,15,16] as well as a higher rate of de novo single nucleotide variants (SNVs) found in exome sequences [17,18] have been observed in females with ASD (f-ASD) than in male cases, especially non-sense and splice site [19,20]. Conversely, a more recent study pointed to sex-specific mutations, specifically on the X chromosome, that may contribute to male prevalence in ASD [21]. On the other hand, as far as sex differences in symptom profiles are concerned, some previous studies suggested different phenotypic features in females than in males with ASD [22] like lower IQ [23], more impaired social and/or communicative functioning [24], psychopathological problems [25] and milder restricted and repetitive behaviors [26,27,28]. However, this issue remains controversial [29,30,31,32,33]. Females with ASD displayed also a higher rate of co-occurring neurological conditions than ASD males, i.e., microcephaly, developmental regression in socialization, minor neurological and musculoskeletal deficits [34], and epilepsy [35], all pointing to sex differences in genetic backgrounds.

The advent of chromosome microarray analysis (CMA) in clinical practice [36] allows for fast detection and accurate characterization of submicroscopic deletions and duplications (CNVs) of genomic DNA associated with ASD [37,38]. Learning societies and ASD experts recommend CMA as part of the first-line evaluation for individuals with ASD [39,40,41]. However, CMA brings up a higher level of polymorphic genomic rearrangements and the process to attribute causality in complex conditions such as ASD is not easy and straightforward.

This study aims to investigate the frequency of causative CNVs in a single-center cross-sectional idiopathic f-ASD cohort to delineate possible genotype/phenotype associations.

## 2. Methods

We collected the clinical data of a group of 93 females referred consecutively to the Autism Spectrum Disorders Unit of our Children Neuropsychiatry Hospital between 2015 and 2016. The age at the last clinical evaluation ranged from 21 months to 17 years. All participants received a clinical diagnosis of ASD based on the criteria of the Diagnostic and Statistical Manual of Mental Disorders (DSM-5) [1]. All the patients were unrelated.

According to our ASD-screening protocol, neurometabolic conditions and hypoxic-ischemic injury were investigated. All participants were evaluated by an expert clinical geneticist in order to exclude recognizable monogenic syndromes. Prior to this study, each individual had also been tested for the expanded repeat sequences in 5′-UTR of the *FMR1* gene as previously reported [42].

Based on this screening, we excluded two females with a history of perinatal hypoxia and diffuse white matter disease detected on brain magnetic resonance imaging (MRI), and one patient with macrocephaly harboring a pathogenic mutation in *PTEN*. In a single case (patient P11) we analyzed CNVs in spite of her presentation of a low-level somatic mosaicism for a fully-mutated/pre-mutated *FMR1* allele, because the patient’s phenotype could not be fully explained by this genetic condition.

Hence, we tested 90 ASD female individuals for CNVs. Participants were classified as clinically affected by “essential” autism, based on the absence of major congenital abnormalities and major dysmorphism [43,44].

Cognitive evaluation was performed in 87 participants with specific cognitive scales based on the age and the language level. According to the age, children were tested respectively with the Griffiths Mental Development Scale—Revised (GMDS-R) [45], Wechsler Preschool and Primary Scale of Intelligence—third edition (WPPSI, III) [46] or Wechsler Intelligence Scale for Children—IV (WISC, IV) [47]. The evaluation of non-verbal females was performed using the Leiter International Performance Scale-Revised (Leiter-R) [48]. In three participants, the cognitive assessment was not performed because of scarce compliance due to severe autism symptoms.

Clinical assessment of expressive language skills defined females with a complete absence of language (*n* = 27) and a group of “verbal” f-ASD (*n* = 63).

The semi-structured Autism Diagnostic Observation Schedule second edition (ADOS-2) evaluation [49], which provides a measure of autism severity, was available in 67 participants. We recorded the score on the Social Affect (SA) and the Restricted and Repetitive Behaviors (RRB) domains for each proband. Since we used different ADOS modules according to the non-echolalic expressive language level of each patient at the time of the evaluation, we converted the global ADOS scores and the sub-scores of the SA and RRB domains in the corresponding Calibrated Severity Score (CSS) [50,51].

This study was approved by the Pediatric Ethic Committee of Tuscany Region (Italy), and was performed according to the Declaration of Helsinki and its later amendments or comparable ethical standards. All parents or legal representatives signed an informed consent form before the inclusion of their child in the study. The identities of all individuals were omitted.

## 3. Procedure

### 3.1. Genetic Analysis

CMA analyses were performed using the Agilent 8 × 60 K Microarray oligonucleotide platform with a median resolution of 100 Kbp, according to the manufacture’s protocol (Agilent Technologies, Santa Clara, CA, USA). CNV coordinates refer to the Genome Reference Consortium Human Build 37 (GRCh37/hg19).

In each proband, CNVs were confirmed by quantitative polymerase chain reaction (qPCR). Segregation analyses in parental DNA (whenever available) were performed by qPCR. Polymorphic CNVs, based on Database of Genomic Variants data (DGV) [52]), were filtered out.

Non-polymorphic CNVs were classified as “causative” (C-CNVs) or “non-causative” (N-CNVs) according to the American College of Medical Genetics and Genomics (ACMG) guidelines [53]. We considered as “causative”: (i) CNVs encompassing genomic regions or genes associated with ASD or with other neuropsychiatric conditions (i.e., intellectual disability, epilepsy and schizophrenia) in the Online Mendelian Inheritance in Man (OMIM) database [54]; (ii) CNVs containing “high confidence” ASD-genes reported in the Simons Foundation Autism Research Initiative (SFARI) Gene database [55] with a score < 3 or in the Autism Knowledge Base version 2.0 (Autism KB 2.0) database [56] with a score > 16; (iii) CNVs involving “candidate-genes” for ASD either reported in association with autism in literature, or listed in the aforementioned databases and with a SFARI Gene score ≥ 3 or an Autism KB score ≤ 16 (suggestive or “low confidence” candidate-genes). Conversely, CNVs were considered non-causative (N-CNVs) if they have never been associated with ASD or other neurodevelopmental disorders (NDDs). Patients who tested negative for CNVs were classified as “without CNVs” (w-CNVs).

To recognize significantly enriched functional modules, ASD-candidate genes encompassed by C-CNVs were evaluated by bioinformatics tools. A Core analysis run in the Variant Effects Analysis mode through the use of the Ingenuity Pathway Analysis (IPA) software [57] figured out cellular processes related to our gene dataset (21 genes). A functional network encompassing our ASD-candidate genes was generated. Bridging nodes were denoted evaluating both direct and indirect interactions with stringent level of confidence and only related to neurological diseases. Gene ontology (GO) categorization was carried out using ToppGene Suite [58]. The top three ontologies for Molecular Functions and Cellular Component were annotated and statistical significance of GO terms was reported as -log10 (*p*-value).

### 3.2. Statistical Analyses

We used a chi-square test to investigate the association between the CNVs subtype and the type of CNVs (duplication or deletion) and the pattern of inheritance (de novo or inherited, paternal or maternal). A Mann–Whitney test was used to verify if there were any differences in the CNVs burden of the different CNVs subtypes (excluding patient P23 who carried a whole X-chromosome duplication).

We also investigated the phenotype of the individuals with the different CNVs subtypes testing with the chi-square test the association between the CNVs subtype and cognitive (IQ ≤ 70 vs. >70) and language (non-verbal vs. verbal) levels. A Mann–Whitney test was used to ascertain that the groups with different CNVs subtype were matched on age and to verify if there were any differences in the CCS score obtained on the total ADOS and on its AS and RRB domains. In case of statistically significantly differences we compute for *r* score as effect size index. This was interpreted as negligible (*r* < 0.10), small (0.10 ≤ *r* < 0.30), medium (0.30 ≤ *r* < 0.50), or large (*r* ≥ 0.50).

## 4. Results

### 4.1. Chromosome Microarray Analysis (CMA)

We performed CMA in 90 females affected by idiopathic ASD, detecting 35 CNVs (17 duplications and 18 deletions) in 29 (32.2%). Twenty-three participants had one CNV and six carried 2 imbalances. Sixty-one f-ASD were considered w-CNVs (67.8% of the whole group).

**Figure 1:**
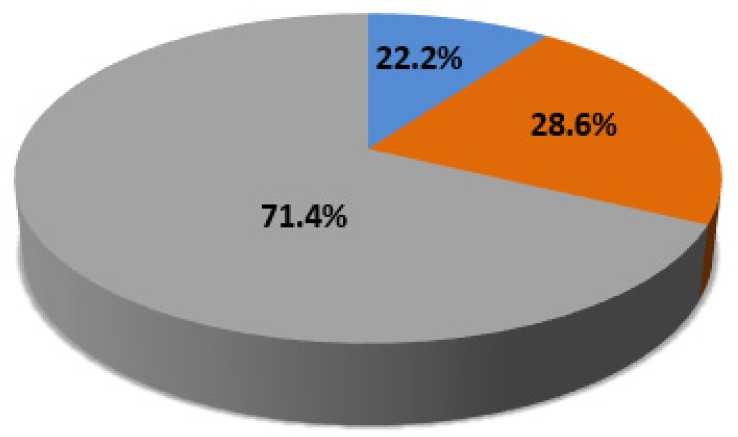
Graphical representation of chromosome microarray analysis (CMA) results in our group of 90 females affected by autism. In the pie chart is depicted the percentage of individuals with causative copy number variants (C-CNVs), non-causative copy number variants (N-CNVs) or without copy number variants (w-CNVs).

Out of 35 CNVs, 25 were classified C-CNVS (71.4%) and 10 N-CNVs (28.6%). In the whole group of 90 f-ASD, 20 patients harbored at least one possible disease-causing CNV (diagnostic yield 22.2%) (Figure 1).

Table 1 illustrates the results of CMA investigations. There were not recurrent C-CNVs, with the exception of two unrelated subjects who harbored a 15q11-q13 microduplication. Ten CNVs involved genomic regions already associated with known contiguous gene-deletion/duplication syndromes associated with ASD or NDDs, 5 CNVs encompassed “high-confident” ASD-genes and ten involved genes reported in literature or in the SFARI Gene/Autism KB databases as possible candidates for autism.

**Figure 2:**
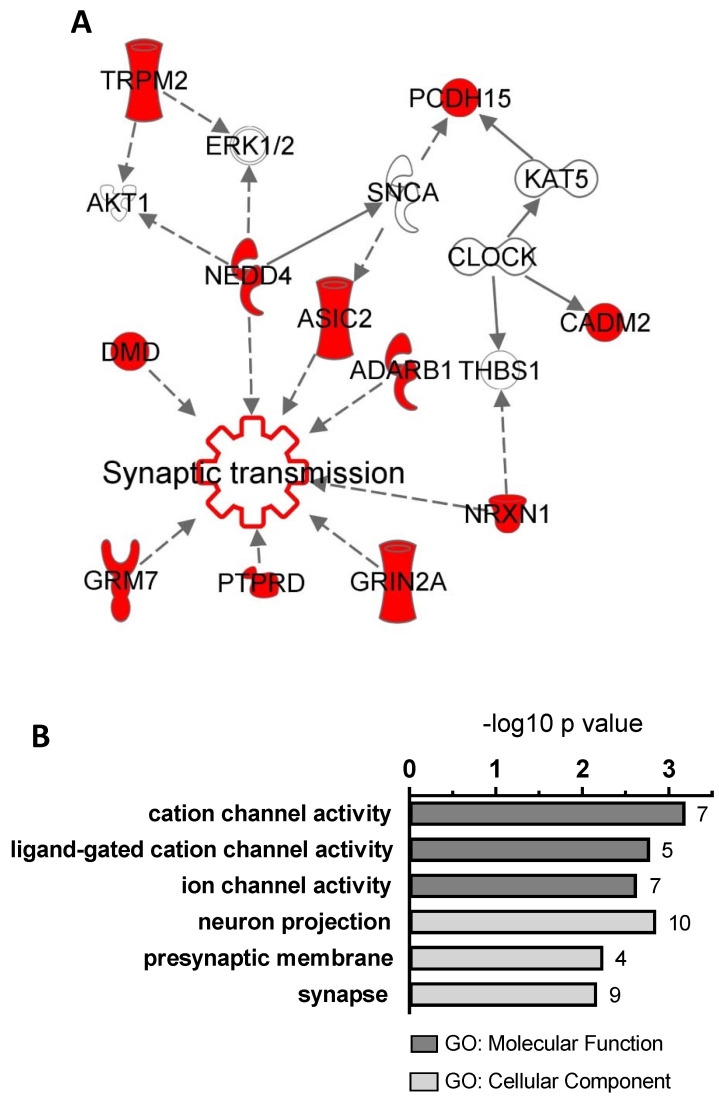
Bioinformatic analyses performed on ASD-candidate genes encompassed by C-CNVs. (**A**) A Core analysis run in Variant Effects Analysis mode using the Ingenuity Pathway Analysis software figured out cellular processes related to our gene dataset (21 genes) generating a functional network encompassing 11 genes (in red). *Synaptic transmission* resulted the most significant functional annotation (*p*-value 6.05 × 10^−9^). Bridging nodes (in white) were denoted evaluating both direct and indirect interactions related only to neurological diseases and with stringent level of confidence (**B**). Gene ontology (GO) categorization was carried out using ToppGene Suite. Top three ontologies for *Molecular Function* (dark grey) and *Cellular Component* (light grey) were annotated; statistical significance of GO terms was reported as −log10 (*p-*value). The number of genes belonging to each category was reported on the right of each bar.

The function and evidence of possible disease-association of the reported candidate-genes are summarized in Table 2. Bioinformatic analysis showed that 11 out of 21 of the reported disease-associated and candidate genes are involved in synaptic structure and transmission (*ADARB1*, *ASIC2*, *CADM2*, *DMD*, *GRIN2A*, *GRM7*, *NEDD4, NRXN1*, *PCDH15*, *PTPRD*, *TRPM2*) (Figure 2).

In 24 f-ASD, carrying 29 CNVs, we assessed a de novo origin in 8 and a paternal in 12, whereas CNVs were maternally-inherited in 9 patients. In 5 children we could not assess segregation because of lack of parental DNA. Table 3 shows the proportion of duplications and deletions and the mode of inheritance in relation to the different subtypes of CNVs. Overall, the rate of de novo CNV was 9.4%. All de novo CNVs involved known NDDs-associated genes/chromosomal regions. CNVs encompassing suggestive or “low confidence” ASD-genes were all inherited; 6 out 9 disrupted more than one NDD-gene or were associated with an additional C-CNV. Seven out of 9 maternally inherited vs. 6 out of 12 paternally inherited CNVs were causative. 

### 4.2. Phenotypic Characterization

Twenty-seven f-ASD had an absence of language whereas 63 were “verbal”.

Cognitive evaluation was performed in 87 participants, being three participants unfit for psychometric testing. Forty-two of the tested individuals had IQ ≤ 70 and 45 had IQ ≥ 70.

The 67 participants tested with ADOS-2 had the following mean (SD) Total, SA and RRB ADOS CSS, respectively: 6.57 (2.36), 6.79 (2.34), and 7.22 (2.30).

Supplementary Table S1 recapitulates clinical data of the studied population.

### 4.3. Statistical Analysis

We observed a statistically significant association between the heritage (de novo vs. maternal and paternal) and the subtypes of CNVs (C-CNVs vs. N-CNVs) (*Chi*^2^_(1)_ = 4.21, *p* = 0.04). Indeed, all N-CNVs were transmitted and never arose de novo while all de novo CNVs were causative (38% of C-CNVs); 7 out of 9 (77.8%) C-CNVs were maternal and 6 out of 12 (50%) were paternal.

Whilst the type of genomic micro-rearrangement (deletion vs. duplication) was not statistically correlated to causative/non-causative definition (*Chi*^2^_(1)_ = 0.41, *p* = 0.52), not considering CNVs associated with contiguous-gene syndromes, most of the breakpoints of causative duplications lie within at least one NDD-candidate gene (*n* = 6/8). C-CNVs had a CNVs burden value statistical significantly higher than those of the N-CNVs subtypes (mean (SD) = 1.14 (1.43) vs. 0.19 (0.16); Mann-Whitney *U* = 52.50, *z* = 2.56, *p* = 0.01, *r* = 0.49).

Table 4 shows the age, the cognitive and linguistic level as well as the autism severity of the three groups of individuals according to different CNV subtypes (causative, non-causative and without CNVs).

To investigate whether there were significant differences in clinical features between groups, we regrouped participants with negative CMA results (N-CNVs and w-CNVs) and compared their characteristics with cases with C-CNVs. The two groups resulted matched for age [mean (SD) = 66.95 (38.55) vs. 56.74 (38.03); Mann–Whitney *U* = 523.00, *z* = 1.72, *p* = 0.09]. 

We found that there were no differences between the two groups on the cognitive level (IQ ≤ 70 vs. IQ > 70; *Chi*^2^_(1)_ = 0.47, *p* = 0.49), language level (non-verbal vs. verbal; *Chi*^2^_(1)_ = 0.31, *p* = 0.58), and on the CSS obtained on the total score and on the AS ADOS domain (Mann–Whitney *U* = 262.50, *z* = 1.42, *p* = 0.16; Mann–Whitney *U* = 303.00, *z* = 0.77, *p* = 0.44).

The relative frequencies of the phenotypic features were the following: in the group with C-CNVs, 55% (11/20) had IQ ≤ 70; 60% had a moderate-severe level of autism symptoms (9/15), 35% had absence of language (7/20); in the group with negative CMA, 46% (31/37) had IQ ≤ 70; 75% had a moderate-severe level of autism symptoms (47/62), 28% had absent language (20/70).

Conversely, we found that the f-ASD with C-CNVs had a statistically significantly lower CSS on the RRB ADOS domain that those without CNVs or with non-causative (mean (SD) = 6.08 (2.14) vs. 7.50 (2.27); Mann–Whitney *U* = 197, *z* = 2.48, *p* = 0.01, *r* = 0.30).

## 5. Discussion

Although a recent meta-analysis and multidisciplinary consensus statement proposes exome sequencing at the beginning of the evaluation of unexplained neurodevelopmental disorders [73], CMA is still the recommended first-tier genetic analysis in the evaluation of ASD subjects [40,74].

In the last few years, investigations of large cohorts of ASD individuals [13,37,75] have identified a high burden of CNVs with rare C-CNVs being found in 5–10% of idiopathic ASD [76]. However, these data are often affected by gender-bias due to the high M/F ratio in the vast majority of the studies and even more recent investigations addressing type and frequency of C-CNVs did not allow—with few exceptions—for separate gender examinations due to relatively small sample size [77,78,79,80].

Herein, we focused exclusively on a cohort of f-ASD and we found clinically significant CNVs in about 22% of patients. Few investigations have considered CNVs and clinical features in f-ASD in comparison with ASD males. In one study, large CNVs (>400 kb) were more frequent in f-ASD than in males (29% vs. 16%), and this difference was even higher (F/M 3:1) if analyses were limited to regions containing genes involved in NDDs [81]. In a similar vein, Levy and colleagues (2011) [13] detected that f-ASD have a high frequency of de novo CNVs (11.7% vs. 7.4% in males), and Sanders et al. (2015) [15] identified a significant difference in the rate of de novo CNVs between boys (5.3%) and girls (8.7%). Our numbers in an only girl cross-sectional, monocentric study denote a similar sex effect with a high diagnostic yield and a 9.4% occurrence of de novo variants.

All de novo CNVs involved known NDDs-associated chromosomal regions whereas CNVs encompassing suggestive or “low confidence” ASD-genes were all inherited and mostly disrupting more than one NDD-gene or associated with an additional C-CNV. Among C-CNVs, there was an excess of maternally-inherited potentially pathogenic CNVs. These findings support the “two-hit model” suggested in previous studies in which the compound effect of a small number of rare variants may contribute to phenotypic heterogeneity of ASD [82].

While literature in the ASD field reported an excess of clinically-significant deletions, we did not find a correlation between the type of genomic rearrangement and causative/non causative definition. Haploinsufficiency for genes within a deletion is a well-recognized cause of genetic disease. Conversely, interpreting the phenotypic consequences of microduplications is often challenging because the pathogenicity of most duplications cannot be explained by triplosensitivity. Sequencing the breakpoints of 119 duplications, Newman et al. (2015) demonstrated that, rather than an extra copy effect, the phenotype of microduplications can be related to the misregulation of genes that span the breakpoints, through loss-of-function mechanisms due to altered transcription or translation or to the creation of fusion proteins with unknown functions [83]. In our f-ASD cohort, most of the causative non-syndromic duplications breakpoints disrupted at least one NDD-candidate gene, hence we can suppose that the pathogenic phenotype could be caused by similar mechanisms.

Unlike previous literature results [78], we did not find any association between C-CNVs and IQ or language deficits. Analyzing the phenotypic features of females with C-CNVs versus those with negative CMA results, we only observed statistically significantly lower scores on the restricted repetitive behaviors (RRB) ADOS domain in f-ASD with clinically significant variants. Recently, Barone et al. reported more severe autistic symptoms in individuals with C-CNVs [79]. The discrepancies with our data could reflect the diverse characteristics of the studied population, indeed several studies suggested a sex effect on RRB scores, which are reported to be repeatedly lower in female than in male subgroups [28,84,85,86]. Crucially, several lines of evidence suggest that social-communication (SC) and RRB symptom domains are underpinned by different genetic mechanisms. For instance, a recent genome-wide association study demonstrated that the RRB trait “systemizing” is heritable and genetically correlated with autism in the general population and that the SC and RRB domains in autistic subjects show low shared genetics [87]. In particular, the contribution of genetic factors to the RRB domain is sustained by their significative presence on both parents [88] and siblings [89] of probands with ASD. Overall, the impact of C-CNVs on ASD symptoms is still unclear and a recent work highlighted the contribution of environmental factors (i.e., maternal infections during pregnancy) on RRB severity in individuals with CNVs [90]. We can only speculate that we registered lower RRB scores in our f-ASD with positive CMA results because this sample represents the mild-end of a genomic “simple” disorder, while those girls with negative results could reflect the group of f-ASD with “complex” multifactorial etiology, as the largest portion of idiopathic autistic males.

With the exception of two subjects with a 15q11-q13 microduplication, no overlapping CNVs were detected, confirming the high genetic heterogeneity of ASD. Fifteen CNVs involved ASD/NDDs-associated genes or genomic regions already identified, whereas 10 CNVs encompassed genes reported as possible candidates for ASD in literature or in ASD databases (Table 1 and Table 2). The contribution of each CNV to the phenotype of our f-ASD patients is discussed in the Supplementary File S1. Out of this list, some cases appear worth discussing.

The known contiguous-gene deletion/duplication syndromes detected in our cases were associated with a diagnosis of “idiopathic” ASD because these patients did not display any of the additional non-neurodevelopmental features specific of these syndromes, as dysmorphisms or congenital defects which can be seen in Smith-Magenis (P8), 17q12 microdeletion (P10), 2p15p16 deletion (P19), 22q11 duplication (P28) and SHOX duplication (P29) syndromes. These patients could represent the mild-end of the phenotypic spectrum of these genomic disorders, due to the “NDDs-protective effect” reported in females [16].

In some cases, reverse phenotyping allowed the investigation and prevention of important comorbidities, as in P25, who carries a de novo partial duplication of the *DMD* gene, which in females could manifest with muscle weakness and cardiomyopathy, and in P20, who carries a 16p11.2 duplication widely reported in ASD studies which is associated with the risk of developing psychotic symptoms [91].

Among clinically relevant rearrangements, aneuploidy was identified in a single subject, who presented an X chromosome trisomy (47, XXX). Interestingly, data in the literature did not report a greater risk for autism in X chromosome trisomy [92], even if difficulties in social functioning and, more broadly, an increased vulnerability for autistic traits are described [68].

The de novo 16p13 duplication detected in one patient (P3) involves partially *UPS7*. Variants affecting this gene were recently reported in 23 individuals with syndromic Developmental Delay/Intellectual Disability [93], and about half of reported subjects had ASD. P3 presents mild motor developmental delay, absent speech, behavioral anomalies and ASD, suggesting that *USP7* haploinsufficiency should be suspected in a case of ASD with absence of speech and behavioral disorders. CNVs detected in P3 spans also *GRIN2A* and *RBFOX1*, so we cannot exclude a possible additional role of these genes in the phenotype of the patient.

The deletions found in P11, P14 and P15 reinforce the evidence of a possible contribution of *PCDH15*, *GRM7*, *CADM2* and *IMMP2L* genes to ASD susceptibility.

Finally, five CNVs spanned some “low-confidence” ASD-genes, which can be investigated in future studies (i.e., *TRPM2*, *ADARB1*, *RFX7*, *NEDD4*, *ASIC2*, *PTPRD*, *ST6GAL2*).

When new and old genes pinpointed by CMA studies were combined in functional modules using IPA and ToppGene Suite, we observed an enrichment in genes involved in synaptic function and transmission, which are well-established biological processes involved in autism and NDDs [94].

In conclusion, this study provides a representative picture of the spectrum of CNV in f-ASD investigated in a clinical setting. As expected, no specific CNVs have been found to be required for developing ASD, supporting the heterogeneity of affected molecular pathways. However, genes in the C-CNVs of our sample of f-ASD code mainly for proteins that could be grouped in two different functional systems: synaptic function/structure, and mRNA/protein processing. Of note, environmental exposures during specific windows of vulnerabilities in prenatal and perinatal life critically interact with genetic susceptibility contributing to ASD pathogenesis [95]. Our study suggests that females with idiopathic ASD have a high rate of pathogenic CNVs encompassing both known and new candidate ASD genes. Hence, studies on large samples of f-ASD carefully assessed from a clinical point of view could help in unraveling the genetic determinants of autism. Moreover, f-ASD with normal-array comparative genomic hybridization analysis could benefit from whole exome or genome sequencing [96], paving the way for the implementation of personalized treatments based on genetic findings.

## Figures and Tables

**Table 1:** Chromosomal microarray (CMA) results in the 29 participants carrying at least one Copy Number Variant (CNV). For each participant with positive CMA results are reported the genomic location and breakpoints of each CNV, the CNV subtype (deletion or duplication), the size in base pairs, the inheritance status, the associated known genetic syndrome or Autism Spectrum Disorders (ASD) candidate genes involved in the rearrangement, and the CNV classification (causative or non-causative).

ID.	CNV Breakpoints	CNV Type	Size (bp)	Inheritance	Syndrome/Candidate Gene	CNV Class	Reference
P1	22q13.33 (50781138-51219009)	del	437,871	de novo	Phelan-McDermid syndrome	C	#MIM 606232
	Xp11.4 (38491539-38628756)	dup	137,217	mat	*TSPAN7*	C	#MIM 300210
P2	14q32.13 (94817951-94883978)	del	66,027	-	-	N	-
P3	16p13.3 (6881091-7070689)	del	189,598	mat	*RBFOX1*	C	[59]
	16p13.2 (9015110-10321593)	dup	1,306,483	de novo	*USP7*, *GRIN2A*	C	[60]
P4	21q22.3 (45822805-46530451)	dup	707,646	mat	*ADARB1*, *TRPM2*, *ITGB2*, *SUMO3*	C	[61]
P5	1q31.2 (191644543-191775583)	del	131,040	pat	-	N	-
P6	2q34.3 (214919902-215051057)	del	131,155	mat	-	N	-
P7	1q21.2 (147211160-147824207)	dup	613,047	pat	-	N	-
P8	17p11.2 (16822483-20193310)	del	3,370,827	de novo	Smith-Magenis syndrome	C	#MIM 182290
P9	15q21.3 (56283008-56384604)	del	101,596	mat	*NEDD4*, *RFX7*	C	[62]
P10	17q12 (34851537-36168104)	del	1,316,567	de novo	17q12 deletion syndrome	C	#MIM 614527
P11	10q21.1 (55616917-55791973)	del	175,056	mat	*PCDH15*	C	[63]
P12	4q34.1 (172930618-173074943)	dup	144,325	pat	-	N	-
	4q34.2 (176984739-177190235)	dup	205,496	pat	-	N	-
P13	17q12 (31953228-32922965)	dup	969,737	mat	*ACCN1, TMEM132E*	C	[64]
P14	3p12.1 (85615568-85672801)	del	57,233	pat	*CADM2*	C	[32]
	3p26.1 (7353126-7403750)	del	50,624	pat	*GRM7*	C	[65]
P15	7q31.1 (110954950-111202026)	del	247,076	pat	*IMMP2L*	C	[66]
P16	2q23.3 (153898093-154164672)	del	266,579	pat	-	N	-
P17	5q23.3 (129687092-130006500)	del	319,408	mat	-	N	-
P18	15q11.2q13.1 (23669701-28525460)	dup	4,825,759	-	15q11q13 duplication syndrome	C	#MIM 608636
P19	2p16.1p15 (58566616-61546442)	del	2,979,826	de novo	2p16.1p15 deletion syndrome	C	#MIM 612513
P20	16p11.2 (29673954-30197341)	dup	523,387	-	16p11.2 duplication syndrome	C	#MIM 614671
P21	15q11.2q13.1 (23669701-28525460)	dup	4,855,759	mat	15q11-q13 duplication syndrome	C	#MIM 608636
P22	9p24.1 (7800020-8528849)	dup	728,829	-	*PTPRD*	C	[61]
	Xp22.31 (6552712-8115153)	del	1,562,441	-	Xp22.31 deletion syndrome	C	[67]
P23	2p16.3 (48915312-48979903)	del	64,591	pat	-	N	-
P24	Xp22.33q28 (61529-155190083)	dup	155,128,554	de novo	47, XXX	C	[68]
P25	Xp21.1 (31893344-32289012)	dup	395,668	de novo	*DMD*	C	[69]
P26	8q24.3 (146053353-146174033	dup	120,680	-	-	N	-
P27	2q12.2q12.3 (106929257-108403252)	dup	1,473,995	pat	*ST6GAL2*	C	[70]
P28	22q11.21 (20754422-21440514)	dup	686,092	pat	22q11.2 duplication syndrome	C	#MIM 608363
P29	2p16.2 (50909765-51083469)	del	173,704	pat	*NRXN1*	C	[71]
	Xp22.33 (581803-920279)	dup	338,476	de novo	*SHOX*	C	[72]

Pt: participant; CNV: copy number variant; bp: base pairs; del: deletion; dup: duplication; mat: maternal; pat: paternal; C: causative; N: non-causative.

**Table 2:** Function and evidences of disease-association of the reported candidate-genes encompassed in causative- Copy Number Variants (CNVs). The table reports evidences that supports the possible role in autism of the reported “high confidence” autism spectrum disorder (ASD) genes (genes with a Simons Foundation Autism Research Initiative SFARI Gene score < 3 or with an Autism KB 2.0 score > 16), and suggestive or “low confidence” candidate-genes (genes with a SFARI Gene score ≥ 3 or with an AutismKB 2.0 score ≤ 16). For each gene, genomic region, participant ID, function of the encoded protein and scores assigned in the SFARI Gene and AutismKB 2.0 databases are reported (NR: gene not reported in the database).

Gene	Genomic Region (Participant ID)	Protein Function	SFARI Gene/AutismKB 2.0
*“High confidence” ASD-genes*			
*USP7*	16p13.2 (P3)	Ubiquitin-specific protease; regulates ubiquitination processes	2/4
*GRIN2A*	16p13.2 (P3)	Subunit 2A of the glutamate N-Methyl-D-Aspartate (NMDA) receptor	2/10
*RBFOX1*	16p13.3 (P3)	RNA-binding protein that regulates alternative splicing events	2/28
*DMD*	Xp21.1 (P25)	Component of the dystrophin-glycoprotein complex (DGC), which bridges the inner cytoskeleton and the extracellular matrix	S/28
*SHOX*	Xp22.33 (P29)	Belongs to the paired homeobox family, nuclear transcription factors involved in cell-cycle and growth regulation	2/2
*NRXN1*	2p16.2 (P29)	Cell adhesion molecule, form a complex with neuroligins at synapses in the central nervous system required for neurotransmission and involved in the formation of synaptic contacts.	1/68
*Suggestive or “low confidence” candidate ASD-genes*			
*TSPAN7*	Xp11.4 (P1)	Member of the tetraspanin family, encodes a cell surface glycoprotein that complex with integrins. It may have a role in neurite outgrowth and Alpha-amino-3-hydroxy-5-methyl-4-isoxazolepropionate (AMPA) receptor trafficking	3/2
*ITGB2*	21q22.3 (P4)	Integrin B2, adhesion molecule implicated in synaptic pruning	NR/3
*TRPM2*	21q22.3 (P4)	Voltage-independent cation channel, mediates sodium and calcium ion influx in response to oxidative stress; modulates oxytocin release.	NR/11
*ADARB1*	21q22.3 (P4)	Protein involved in the editing of the RNA of glutamate, serotonin and Gamma-Aminobutyric Acid (GABA) receptors, and potassium voltage-gated channels.	5/1
*SUMO3*	21q22.3 (P4)	Involved in SUMOylation of proteins, a post-translational modification that modulates the activity of several neuronal transcription factors	NR/0
*RFX7*	15q21.3 (P9)	Transcription factor	NR/4
*NEDD4*	15q21.3 (P9)	Protein involved in the ubiquitin proteasome system. It plays a critical role in the ubiquitination and degradation of AMPA receptors, endocytic machinery components and Phosphatase and Tensin Homolog (PTEN) protein.	NR/4
*PCDH15*	10q21.1 (P11)	Member of the cadherin superfamily, membrane proteins that mediate cellular adhesion	3/16
*ACCN1 (ASIC2)*	17q12 (P13)	Non-voltage-dependent Na^+^ channel; facilitates Acid-Sensing Ion Channel (ASIC) localization to synapses interacting with synaptic scaffolding proteins as Postsynaptic Density Protein 95 (PSD95)	NR/7
*TMEM132E*	17q12 (P13)	Neural adhesion molecule	NR/NR
*CADM2*	3p12.1 (P14)	Adhesion molecule involved in synapse organization, providing regulated trans-synaptic adhesion.	3/0
*GRM7*	3p26.1 (P14)	Metabotropic glutamate receptor mGluR7	3/12
*IMMP2L*	7q31.1 (P15)	Subunit of an inner mitochondrial membrane peptidase complex involved in processing of mitochondrial proteins	3/10
*PTPRD*	9p23p24 (P22)	Receptor protein tyrosine phosphatase, induces pre- and post-synaptic differentiation and regulates neurogenesis. Interacts with proteins involved in intellectual disability/ASD as IL1RAP and IL1RAPL1 and proteins of the mitogen-activated protein kinase (MEK)/extracellular signal-regulated kinase (ERK) pathway.	NR/7
*ST6GAL2*	2q12.3 (P27)	Encodes a sialyltransferase mostly expressed in embryonic and adult brain. CNVs were reported in autism studies.	NR/2

**Table 3:** Proportion of deletions vs. duplications and pattern of inheritance of the reported CNVs according to their classification (causative vs. non-causative).

	Type of CNVs		Inheritance		
	Duplication	Deletion	De novo	Paternal	Maternal
	(*n* = 17)	(*n* = 18)	(*n* = 8)	(*n* = 12)	(*n* = 9)
C-CNVs	13/25 (52%)	12/25 (48%)	8/21 (38.1%)	6/21 (28.6%)	7/21 (33.3%)
(*n* = 25)					
N-CNVs	4/10 (40%)	6/10 (60%)	0/8 (0%)	6/8 (75%)	2/8 (25%)
(*n* = 10)					
Total	17/35 (48.6%)	18/35 (51.4%)	8/29 (27.6%)	12/29 (41.4%)	9/29 (31%)

*Note:* Inheritance was assessed in 29 out of 35 CNVs. C-CNVs = causative CNVs; N-CNVs = non-causative CNVs; *n* = number of CNVs for each group.

**Table 4:** Demographic features of participants grouped according to CMA results. For each group (with causative and non-causative CNVs, or without CNVs) are reported the mean age at the last examination (in months), the rate of patients with a IQ level > 70 vs. ≤70, the rate of verbal vs. non-verbal patients, and the mean calibrated severity scores (CSS) of the global Autism Diagnostic Observation Schedule (ADOS) scores and the sub-scores of the Social Affect (SA) and Restricted and Repetitive Behaviors (RRB) domains. The language level was assessed in all 90 participants, the IQ level and the ADOS scores were available for 87 and 67 of the 90 individuals, respectively.

	C-CNVs	N-CNVs	w-CNVs
	(*n* = 20)	(*n* = 9)	(*n* = 61)
Mean age at the last examination in months (SD)	66.95 (38.55)	47.11 (15.57)	58.16 (40.19)
IQ > 70	9/20 (45%)	4/8 (50%)	32/59 (54.2%)
IQ ≤ 70	11/20 (55%)	4/8 (50%)	27/59 (45.8%)
Verbal	13/20 (65%)	5/9 (55.6%)	45/61 (73.7%)
Non-verbal	7/20 (35%)	4/9 (44.4%)	16/61 (26.3%)
Mean ADOS-CSS:	-	-	-
Mean SA-CSS (SD)	6.38 (2.26)	7.50 (1.64)	6.81 (2.45)
Mean RRB-CSS (SD)	6.08 (2.14)	5.50 (3.83)	7.75 (1.92)
Mean Global-CSS (SD)	5.69 (2.25)	6.83 (2.64)	6.77 (2.35)

C-CNVs = participants with causative CNVs; N-CNVs = participants with non-causative CNVs; w-CNVs = participants without; *n* = number of patients for each group; SD = standard deviation.

## Supplementary Materials

The following are available online at https://www.mdpi.com/2075-4426/10/4/160/s1, Table S1: Phenotipic characteristics of participants; Supplementary File S1: Contribution of each CNV to the phenotype of f-ASD patients.
