# Supplementary material for: Evaluation of Chromosome Microarray Analysis in a Large Cohort of Females with Autism Spectrum Disorders: A Single Center Italian Study
Source: J Pers Med. 2020 Oct 9;10(4):160. doi: 10.3390/jpm10040160 (PMC7720139; doi:10.3390/jpm10040160)
Supplement: Supplementary file 1 [file jpm-10-00160-s001.zip › rev_Supplementary File S1.docx]

**Supplementary File S1**

Contribution of each CNV to the phenotype of f-ASD patients.

**CNVs encompassing known genomic disorders or strong ASD-candidate genes**

***Patient P1***

The *de novo* 22q13.33 deletion spanning about 430 kb detected in P1 involves, among others, the SH3 and multiple ankyrin repeat domains 3 (*SHANK3*) gene that encodes a key protein for the regulation of excitatory synapses structure and function [1]. *SHANK3* mutations have been implicated in ~1% of all individuals with ASD [2]. Deletion involving this gene are responsible for the Phelan-McDermid syndrome (PMS) whose neuropsychiatric manifestations include also intellectual disability, epilepsy, hypotonia, severe impairment in speech, as well as bipolar disorder and cognitive/behavioral regression with increasing age [3]. A direct correlation between the deletion size and the severity of PMS symptoms was reported [4].

In addition, P1 carries an interstitial Xp11 duplication that disrupts the *TSPAN7* gene (MIM 300096), whose mutations cause a non-syndromic X-linked recessive form of mental retardation (MIM 300210). This duplication is inherited from a typically developing mother. P1 presents ID and severe language delay. This features are consistent with PMS but we cannot exclude a contribution of the Xp11 duplication to the phenotype.

***Patient P3***

P3 has a *de novo* duplication, which involves two genes already associated with neuropsychiatric conditions, *GRIN2A* (MIM 138253) and exon 1-4 of *USP7* (MIM 602519). Three partially overlapping duplications were reported in subjects with ASD [5]. *GRIN2A* codes for a subunit of the glutamate NMDA receptor. Intragenic mutations and deletions of this gene cause an epileptic syndrome with speech disorder and ID/ASD/ADHD in some cases (MIM 245570). *USP7* is an ubiquitin-specific protease that regulates the ubiquitination of many proteins. *USP7* haploinsufficiency was recently associated to a novel neurodevelopmental disorder. Almost all reported individuals had speech delay (18% were non-verbal), DD/ID and non-specific facial dysmorphisms. Frequently associated features were behavioral anomalies (57%), ASD (53%), ADHD (38%), eye anomalies (65%), hypotonia (67%), MRI anomalies (73%), seizures (45%) and hypogonadism (44%) [6].

In this patient CMA detected an additional maternal deletion involving intron 3 of *RBFOX1* (MIM 605104), which encodes a RNA-binding protein that regulates alternative splicing events of several genes crucial for neurodevelopment. Overlapping inherited deletions were reported in two individuals with ASD, both carried an additional imbalance inherited from the other parent and an oligogenic model for ASD was suggested [7].

Interestingly, P3 shares some features with the reported patients with *USP7* haploinsufficiency, as developmental delay, absent speech, autism, emotional dysregulation and attention deficits, mild intermittent esotropia and hypotonia. She did not displayed dysmorphic features and EEG was nomal. We considered the partial duplication of *USP7* as pathogenic due to the *de novo* occurrence and the literature data. However, we cannot exclude a possible additional role of the *GRIN2A* duplication and of the *RBFOX1* intronic deletion in the phenotype.

***Patient P8***

The interstitial *de novo* deletion in chromosome 17p11.2 detected in P8 is known to be responsible for the Smith-Magenis syndrome (SMS) (MIM 182290). Approximately 35% of individuals with SMS score above the cutoff for ASD [8], and females seem to present a more severe ASD phenotype, particularly in the social domain [9]. P8 presents the behavioral manifestations of SMS as self-destructive behaviors and hand-biting, but she didn’t displayed other syndromic features, as dimorphisms and congenital anomalies.

***Patient P10***

P10 has a deletion overlapping the 17q12 microdeletion syndrome region (MIM 614527). Patients with deletion of this chromosomal band share several features, including genitourinary and renal defects, facial dimorphisms, ID, speech delay and ASD. P10 did not present dismorphic features or congenital defects.

***Patients P18 and P21***

An interstitial microduplication involving the 15q11q13 duplication syndrome region (MIM 608636) is the only recurrent CNV in our f-ASD sample, since it has been detected in two different subjects (P18 and P21). This imbalance is due to non-allelic homologous recombination (NAHR) events between segmental duplications. In both patients the duplication overlaps the Prader–Willi/Angelman syndrome critical region (BP2-BP3 genomic interval). Duplications involving the maternal 15q11q13 region are characterized by a variable phenotype, ranging from mild developmental delay and autism to severe phenotypes of intellectual and developmental delay, seizures, autism and minor, non-specific facial features [10]. A study from Autism Genome Project analyzing 2446 ASD-affected families found that the most frequent duplication is 15q11-13, which was identified in 0.25% of affected siblings [11].

P18 shows moderate ASD symptoms with normal cognitive and language skills. The EEG revealed bilateral focal epileptic discharges but she does not suffer of clinical seizures.

P21 presented with ASD and mild developmental and language delay. EEG was normal. Her younger sister carries the same 15q duplication; she displays autistic features, language delay and epileptic anomalies at the EEG. The 15q duplication was inherited from a non-affected mother.

***Patient P19***

In P19 is affected by the *2p16.1p15 deletion syndrome* (OMIM 612513). This syndrome manifests with an extensive range of clinical manifestations, including different degrees of intellectual disability, language impairment, autistic symptoms, microcephaly, neuromotor deficits, strabismus, dysmorphic features and several congenital anomalies (i.e. valvular, renal and brain defects) [12]. P19 shows moderate ID and ASD; congenital malformations were excluded and brain MRI was normal.

***Patient P20***

The chromosomal duplication in the 16p11.2 region detected in P20 has been widely reported in ASD studies [13]. A meta-analysis of 3613 ASD subjects reported that deletion and duplication in 16p11.2 may account for approximately 0.76% of cases [14].

This syndromic condition (MIM 614671) is associated with high rates of psychopathology (especially ASD, ADHD, psychotic symptoms, intellectual disability) [15], low body mass index (BMI), and reduced head circumference [16]. Interestingly, reviewing the phenotype, at the age of 10 years P20 presented auxological parameters in the low-normal range (weight, height and head circumference at 10^th^ percentile) and some psychotic symptoms.

***Patient P22***

The Xp22.31 deletion found in patient P22 is associated to several NDDs (specifically, ID, ASD and epilepsy), mainly in males but recurrent Xp22.31 deletions and duplications are reported also in females with variable phenotypes. Recently this imbalances were identified as risk factors in females with rolandic epilepsy [17]. In our patient EEG revealed some diffuse epileptiform discharges during sleep.

P22 carries an additional 9p duplication that involves part of the *PTPRD* gene, which encodes a receptor protein tyrosine phosphatase, which has been associated with ASD [18]. The function of PTPRD gene in central nervous system involves a number of brain developmental processes which are disrupted in ASD, including cell growth, proliferation, and migration, axon guidance and synaptogenesis [19,20].

***Patient P22***

Patient P22 has a X chromosome trisomy. Sex chromosomes aneuploidies such as Klinefelter, 47,XYY and Turner syndrome confer a greater risk of ASD [21,22]. Conversely, literature did not report a greater risk for autism in subjects with X chromosome trisomy [23], even if difficulties in social functioning and, more broadly, an increased vulnerability for autistic symptomatology are described [24].

***Patient P25***

P25 carried a *de novo* duplication involving exons 44-48 of the *DMD* gene (MIM 300377), encoding dystrophin. Some dystrophin isoforms are highly expressed in human cerebral cortex and cerebellum and several studies suggest a relationship between mutations downstream exon 45, which specifically affects the brain-specific isoform dp140, with the cognitive and behavioural phenotype of Duchenne muscular dystrophy (DMD) [25]. DMD patients have greater incidence of hyperactive features, behavioural problems, learning difficulties/ID. Up to 19% of DMD males has autistic symptoms. Several studies on the cognitive phenotype dystrophinopathies outlined a peculiar profile in which language and speech are more affected than non-verbal skills [26] [26,27]. One third of symptomatic DMD female carriers present intellectual disabilities or behavioural issues, although a formal diagnosis of ASD is rare [28].

Reviewing the phenotype, P25 presented mild autistic symptoms, a low-average development and main difficulties in language and speech with apraxic deficits. The neurological examination, performed by a clinician expert in muscular disorders after the genetic results, detected only mild calf hypertrophy. CK levels were high (up to 1921 U/L; normal <170).

***Patient P28***

P28 has a paternal duplication involving the “central” 22q11.2 region. The 22q11 chromosomal band encompasses several segmental duplications that facilitates NAHR events. According to the possible different breakpoints (BPs), NAHR can lead to several CNVs, both microdeletions and microduplication, involving the “proximal” (BPs A-B), “central” (BPs B-D) and “distal” (BPs D-h) regions respectively. 22q11.2 microduplications are associated with a wide range of neuropsychiatric manifestations. A recent study identified ASD in 14-25% of subjects with this chromosomal imbalance, and some features of ASD or other neuropsychiatric problems in an additional 30% of cases. To date, few cases with central 22q11.2 duplications have been reported with variable phenotypes. These duplications are usually inherited from a healthy parent, as in P28, and could be a possible risk factor for neurodevelopmental disorders, particularly for ASD, speech delay and behavioral abnormalities [29]. Moreover, individuals with 22q11.2 central duplications can be affected by some comorbidities typically associated with the *22q11.2 duplication syndrome* (MIM 608363), including heart septal defects disease, palatal clefts and growth delay, that were excluded in our patient.

***Patient P29***

The *de novo* chromosome Xp22.3 duplication detected in P29 encompasses the *SHOX* gene (short stature homeobox-containing gene), which encodes a nuclear transcription factor involved in cell-cycle and growth regulation and is known to play an important role in the final height determination [30]. Microduplications affecting *SHOX* are considered pathogenic variants with reduced penetrance and heterogeneous phenotypic expressivity. A statistically significant enrichment of *SHOX* microduplications was demonstrated in NDD cases, particularly in those with ASD, compared with controls [31]. Literature suggests that a considerable proportion (16.5%) of patients with *SHOX* microduplications also carries additional CNVs. In line with the ‘two-hit hypothesis’ [32], the additional imbalances could act in synergy with the *SHOX* duplication causing the autistic phenotype. This is the case of our patient. She carries also a 2p16.2 paternal deletion involving exons 1-5 of the *NRXN1* gene (MIM 600565). This gene encodes for a presynaptic adhesion molecule. Deletions in the first five exons of *NRXN1* are incomplete penetrance CNVs associated mainly with ASD/psychiatric conditions with or without mild ID/DD or borderline IQ [33]. The neurodevelopmental phenotype of P29 could be the result of the compound effect of both CNVs. Moreover, SHOX rearrangements are associated with variations in height [34]. At 4 years and 5 months, she presented stature above +2 SD (117 cm = +2.44 SD), with head circumference and weight in the normal range (50.5 cm = +0.12 SD and 18.7 kg = +0.62 SD, respectively).

**CNVs encompassing suggestive and “low-confidence” ASD-candidate genes**

***Patient P4***

P4 carries a maternal 21q22 duplication spanning, among others, 3 brain-expressed genes: *SUMO3* (MIM 602231), *ITGB2* (MIM 600065), the terminal portion of *TRPM2* (MIM 603749) and the first exon of *ADARB1* (MIM 601218). SNPs in *TRPM2* were reported as risk factors in bipolar disorder and ASD [35]. *ITGB2* codes for an adhesion molecule implicated in synaptic connectivity. This gene is already associated to a form of immune dysfunction (MIM 116920). Analyses on ADS brain samples detected a hypometilation of this gene [36]. Moreover, paralogs of *TRPM2* and *ITGB2* are still reported as possible ASD-candidates. *ADARB1* codes for a FMRP-interacting protein involved in RNA-editing of AMPA glutamate receptors and other synaptic proteins. Recently, biallelic missense variants of *ADARB1* where found in patients affected by epileptic encephalopathy, microcephaly and severe ID [37].

***Patient P9***

P9 has a maternal deletion located in 15q21 that encompasses the first exon of *NEDD4* (MIM 602278) and the terminal portion of *RFX7* (MIM 612660). Both genes are highly expressed in brain. *NEDD4* produce an ubiquitin-ligase that interacts with PTEN and FMRP. Several studies suggest that Nedd4 is responsible for AMPAR ubiquitination, regulating their localization and stability [38]. Its paralog NEDD4L is involved in epilepsy and neuronal migration defects [39]. *RFX7* encodes a transcription factor that plays an important role in the development of the neural tube during embryogenesis [40]. This transcription factor interacts with *CHD8*, a gene associated with autism that modulates PTEN pathway. Its paralog *RFX3* has been listed in in the SfariGene database as a “high-confidence” ASD gene.

***Patient P11***

P11 harbours a deletion in the maternal 10q21 chromosomal region, involving part of *PCDH15* (exon 21-30) (MIM 605514), which is a member of the cadherin super-family, proteins that mediate cellular adhesion and neural development and function. Recessive mutations in this gene are associated to deafness (MIM 609533) and Usher syndrome type 1 (USH1, MIM 602083). *PCDH15*-associated USH1 (MIM 601067) has also digenic inheritance. Rare exonic and single-nucleotide variants in *PCDH15* were identified in individuals with ASD and schizophrenia (SCZ) [41]. P11 has also a low-level *FMR1* mosaic permutated/fully mutated allele.

***Patient P13***

P13 has a maternal duplication involving the first exon of *ACCN1/ASIC2* (MIM 601784)*.* This gene encodes an acid sensing ion channel which facilitates ASIC channel localization to synapses interacting with the neuronal synaptic scaffolding protein PSD95, whose mutations cause intellectual disability (MIM 618793). Genome-wide studies have associated variants involving *ASIC2* with autism, psychiatric disorders and dyslexia [42,43]. The duplication disrupts also *TMEM132E*, which codes for a highly conserved protein that belongs to a new family of neural adhesion molecules. Variants in this gene are associated to panic disorder and autosomal recessive non-syndromic hearing impairment [44].

***Patient P14***

P14 carries a paternal deletion involving intron 4 of the *GRM7* gene (MIM 604101), encoding the metabotropic glutamate receptor 7 (mGluR7). Structural variants in mGluR signalling pathways are significantly enriched in ASD, ADHD and SCZ [45]. P14 also has an additional paternal intronic deletion in *CADM2* (MIM 609938), which encodes a synaptic cell adhesion molecule important in the early postnatal development of the CNS. Variants in this gene have a weak positive association with ASD [46].

***Patient P15***

P15 has a paternal deletion of exons 2-4 of the *IMMP2L* gene (MIM 605977), encoding a subunit of an inner mitochondrial membrane peptidase complex involved in processing of mitochondrial proteins. *IMMP2L* is a possible susceptibility gene in Gilles de la Tourette syndrome (GTS) and attention deficit hyperactivity disorder (ADHD), two conditions with clinical and genetic overlap with ASD. The *Immp2l* knockdown mouse model display both gene-dose and sex-dependent behavioural alterations, with a more relevant reduction in social behaviour in heterozygous females. In humans, *IMMP2L* disruption has been correlated with ASD but this association is still debated [47].

Several imbalances involving *IMMP2L* are reported in the DECIPHER dataset in patients with NDDs. The penetrance of these defects is not complete since they often are inherited from a healthy parent.

***Patient P27***

In P27 was found a paternal duplication in 2q12 that contains *ST6GAL2* (MIM 608472). This gene encodes a sialyltransferase mostly expressed in embryonic and adult brain. Some evidences suggest that *ST6GAL2* could be implicated in ASD and schizophrenia [48–50].

**References**

1. Wang, X.; Xu, Q.; Bey, A.L.; Lee, Y.; Jiang, Y.-H. Transcriptional and functional complexity of Shank3 provides a molecular framework to understand the phenotypic heterogeneity of SHANK3 causing autism and Shank3 mutant mice. *Mol. Autism* **2014**, *5*, 30, doi:10.1186/2040-2392-5-30.

2. Uchino, S.; Waga, C. SHANK3 as an autism spectrum disorder-associated gene. *Brain Dev.* **2013**, *35*, 106–10, doi:10.1016/j.braindev.2012.05.013.

3. Kohlenberg, T.M.; Trelles, M.P.; McLarney, B.; Betancur, C.; Thurm, A.; Kolevzon, A. Psychiatric illness and regression in individuals with Phelan-McDermid syndrome. *J. Neurodev. Disord.* **2020**, *12*, 7, doi:10.1186/s11689-020-9309-6.

4. Soorya, L.; Kolevzon, A.; Zweifach, J.; Lim, T.; Dobry, Y.; Schwartz, L.; Frank, Y.; Wang, A.T.; Cai, G.; Parkhomenko, E.; et al. Prospective investigation of autism and genotype-phenotype correlations in 22q13 deletion syndrome and SHANK3 deficiency. *Mol. Autism* **2013**, *4*, 18, doi:10.1186/2040-2392-4-18.

5. Sanders, S.J.; Ercan-Sencicek, A.G.; Hus, V.; Luo, R.; Murtha, M.T.; Moreno-De-Luca, D.; Chu, S.H.; Moreau, M.P.; Gupta, A.R.; Thomson, S.A.; et al. Multiple recurrent de novo CNVs, including duplications of the 7q11.23 Williams syndrome region, are strongly associated with autism. *Neuron* **2011**, *70*, 863–85, doi:10.1016/j.neuron.2011.05.002.

6. Fountain, M.D.; Oleson, D.S.; Rech, M.E.; Segebrecht, L.; Hunter, J. V; McCarthy, J.M.; Lupo, P.J.; Holtgrewe, M.; Moran, R.; Rosenfeld, J.A.; et al. Pathogenic variants in USP7 cause a neurodevelopmental disorder with speech delays, altered behavior, and neurologic anomalies. *Genet. Med.* **2019**, *21*, 1797–1807, doi:10.1038/s41436-019-0433-1.

7. Bacchelli, E.; Cameli, C.; Viggiano, M.; Igliozzi, R.; Mancini, A.; Tancredi, R.; Battaglia, A.; Maestrini, E. An integrated analysis of rare CNV and exome variation in Autism Spectrum Disorder using the Infinium PsychArray. *Sci. Rep.* **2020**, *10*, 3198, doi:10.1038/s41598-020-59922-3.

8. Oliver, C.; Berg, K.; Moss, J.; Arron, K.; Burbidge, C. Delineation of behavioral phenotypes in genetic syndromes: characteristics of autism spectrum disorder, affect and hyperactivity. *J. Autism Dev. Disord.* **2011**, *41*, 1019–32, doi:10.1007/s10803-010-1125-5.

9. Nag, H.E.; Nordgren, A.; Anderlid, B.-M.; Nærland, T. Reversed gender ratio of autism spectrum disorder in Smith-Magenis syndrome. *Mol. Autism* **2018**, *9*, 1, doi:10.1186/s13229-017-0184-2.

10. Al Ageeli, E.; Drunat, S.; Delanoë, C.; Perrin, L.; Baumann, C.; Capri, Y.; Fabre-Teste, J.; Aboura, A.; Dupont, C.; Auvin, S.; et al. Duplication of the 15q11-q13 region: clinical and genetic study of 30 new cases. *Eur. J. Med. Genet.* **2014**, *57*, 5–14, doi:10.1016/j.ejmg.2013.10.008.

11. Pinto, D.; Delaby, E.; Merico, D.; Barbosa, M.; Merikangas, A.; Klei, L.; Thiruvahindrapuram, B.; Xu, X.; Ziman, R.; Wang, Z.; et al. Convergence of genes and cellular pathways dysregulated in autism spectrum disorders. *Am. J. Hum. Genet.* **2014**, *94*, 677–94, doi:10.1016/j.ajhg.2014.03.018.

12. Piccione, M.; Piro, E.; Serraino, F.; Cavani, S.; Ciccone, R.; Malacarne, M.; Pierluigi, M.; Vitaloni, M.; Zuffardi, O.; Corsello, G. Interstitial deletion of chromosome 2p15-16.1: report of two patients and critical review of current genotype-phenotype correlation. *Eur. J. Med. Genet.* **2012**, *55*, 238–44, doi:10.1016/j.ejmg.2012.01.014.

13. Fernandez, B.A.; Roberts, W.; Chung, B.; Weksberg, R.; Meyn, S.; Szatmari, P.; Joseph-George, A.M.; Mackay, S.; Whitten, K.; Noble, B.; et al. Phenotypic spectrum associated with de novo and inherited deletions and duplications at 16p11.2 in individuals ascertained for diagnosis of autism spectrum disorder. *J. Med. Genet.* **2010**, *47*, 195–203, doi:10.1136/jmg.2009.069369.

14. Walsh, K.M.; Bracken, M.B. Copy number variation in the dosage-sensitive 16p11.2 interval accounts for only a small proportion of autism incidence: a systematic review and meta-analysis. *Genet. Med.* **2011**, *13*, 377–84, doi:10.1097/GIM.0b013e3182076c0c.

15. Niarchou, M.; Chawner, S.J.R.A.; Doherty, J.L.; Maillard, A.M.; Jacquemont, S.; Chung, W.K.; Green-Snyder, L.; Bernier, R.A.; Goin-Kochel, R.P.; Hanson, E.; et al. Psychiatric disorders in children with 16p11.2 deletion and duplication. *Transl. Psychiatry* **2019**, *9*, 8, doi:10.1038/s41398-018-0339-8.

16. D’Angelo, D.; Lebon, S.; Chen, Q.; Martin-Brevet, S.; Snyder, L.G.; Hippolyte, L.; Hanson, E.; Maillard, A.M.; Faucett, W.A.; Macé, A.; et al. Defining the Effect of the 16p11.2 Duplication on Cognition, Behavior, and Medical Comorbidities. *JAMA psychiatry* **2016**, *73*, 20–30, doi:10.1001/jamapsychiatry.2015.2123.

17. Addis, L.; Sproviero, W.; Thomas, S. V; Caraballo, R.H.; Newhouse, S.J.; Gomez, K.; Hughes, E.; Kinali, M.; McCormick, D.; Hannan, S.; et al. Identification of new risk factors for rolandic epilepsy: CNV at Xp22.31 and alterations at cholinergic synapses. *J. Med. Genet.* **2018**, *55*, 607–616, doi:10.1136/jmedgenet-2018-105319.

18. Gai, X.; Xie, H.M.; Perin, J.C.; Takahashi, N.; Murphy, K.; Wenocur, A.S.; D’arcy, M.; O’Hara, R.J.; Goldmuntz, E.; Grice, D.E.; et al. Rare structural variation of synapse and neurotransmission genes in autism. *Mol. Psychiatry* **2012**, *17*, 402–11, doi:10.1038/mp.2011.10.

19. Muhle, R.A.; Reed, H.E.; Stratigos, K.A.; Veenstra-VanderWeele, J. The Emerging Clinical Neuroscience of Autism Spectrum Disorder: A Review. *JAMA psychiatry* **2018**, *75*, 514–523, doi:10.1001/jamapsychiatry.2017.4685.

20. Tomita, H.; Cornejo, F.; Aranda-Pino, B.; Woodard, C.L.; Rioseco, C.C.; Neel, B.G.; Alvarez, A.R.; Kaplan, D.R.; Miller, F.D.; Cancino, G.I. The Protein Tyrosine Phosphatase Receptor Delta Regulates Developmental Neurogenesis. *Cell Rep.* **2020**, *30*, 215–228.e5, doi:10.1016/j.celrep.2019.11.033.

21. Ross, J.L.; Roeltgen, D.P.; Kushner, H.; Zinn, A.R.; Reiss, A.; Bardsley, M.Z.; McCauley, E.; Tartaglia, N. Behavioral and Social Phenotypes in Boys With 47,XYY Syndrome or 47,XXY Klinefelter Syndrome. *Pediatrics* **2012**, *129*, 769–778, doi:10.1542/peds.2011-0719.

22. Donnelly, S.L.; Wolpert, C.M.; Menold, M.M.; Bass, M.P.; Gilbert, J.R.; Cuccaro, M.L.; Delong, G.R.; Pericak-Vance, M.A. Female with autistic disorder and monosomy X (Turner syndrome): parent-of-origin effect of the X chromosome. *Am. J. Med. Genet.* **2000**, *96*, 312–6, doi:10.1002/1096-8628(20000612)96:3<312::aid-ajmg16>3.0.co;2-8.

23. Bishop, D.V.M.; Jacobs, P.A.; Lachlan, K.; Wellesley, D.; Barnicoat, A.; Boyd, P.A.; Fryer, A.; Middlemiss, P.; Smithson, S.; Metcalfe, K.; et al. Autism, language and communication in children with sex chromosome trisomies. *Arch. Dis. Child.* **2011**, *96*, 954–9, doi:10.1136/adc.2009.179747.

24. van Rijn, S.; Stockmann, L.; Borghgraef, M.; Bruining, H.; van Ravenswaaij-Arts, C.; Govaerts, L.; Hansson, K.; Swaab, H. The social behavioral phenotype in boys and girls with an extra X chromosome (Klinefelter syndrome and Trisomy X): a comparison with autism spectrum disorder. *J. Autism Dev. Disord.* **2014**, *44*, 310–20, doi:10.1007/s10803-013-1860-5.

25. Doorenweerd, N.; Mahfouz, A.; van Putten, M.; Kaliyaperumal, R.; T’ Hoen, P.A.C.; Hendriksen, J.G.M.; Aartsma-Rus, A.M.; Verschuuren, J.J.G.M.; Niks, E.H.; Reinders, M.J.T.; et al. Timing and localization of human dystrophin isoform expression provide insights into the cognitive phenotype of Duchenne muscular dystrophy. *Sci. Rep.* **2017**, *7*, 12575, doi:10.1038/s41598-017-12981-5.

26. Darmahkasih, A.J.; Rybalsky, I.; Tian, C.; Shellenbarger, K.C.; Horn, P.S.; Lambert, J.T.; Wong, B.L. Neurodevelopmental, behavioral, and emotional symptoms common in Duchenne muscular dystrophy. *Muscle Nerve* **2020**, *61*, 466–474, doi:10.1002/mus.26803.

27. Perumal, A.R.; Rajeswaran, J.; Nalini, A. Neuropsychological profile of duchenne muscular dystrophy. *Appl. Neuropsychol. Child* **2015**, *4*, 49–57, doi:10.1080/21622965.2013.802649.

28. Papa, R.; Madia, F.; Bartolomeo, D.; Trucco, F.; Pedemonte, M.; Traverso, M.; Broda, P.; Bruno, C.; Zara, F.; Minetti, C.; et al. Genetic and Early Clinical Manifestations of Females Heterozygous for Duchenne/Becker Muscular Dystrophy. *Pediatr. Neurol.* **2016**, *55*, 58–63, doi:10.1016/j.pediatrneurol.2015.11.004.

29. Woodward, K.J.; Stampalia, J.; Vanyai, H.; Rijhumal, H.; Potts, K.; Taylor, F.; Peverall, J.; Grumball, T.; Sivamoorthy, S.; Alinejad-Rokny, H.; et al. Atypical nested 22q11.2 duplications between LCR22B and LCR22D are associated with neurodevelopmental phenotypes including autism spectrum disorder with incomplete penetrance. *Mol. Genet. genomic Med.* **2019**, *7*, e00507, doi:10.1002/mgg3.507.

30. Seo, G.H.; Kang, E.; Cho, J.H.; Lee, B.H.; Choi, J.-H.; Kim, G.-H.; Seo, E.-J.; Yoo, H.-W. Turner syndrome presented with tall stature due to overdosage of the SHOX gene. *Ann. Pediatr. Endocrinol. Metab.* **2015**, *20*, 110–3, doi:10.6065/apem.2015.20.2.110.

31. Tropeano, M.; Howley, D.; Gazzellone, M.J.; Wilson, C.E.; Ahn, J.W.; Stavropoulos, D.J.; Murphy, C.M.; Eis, P.S.; Hatchwell, E.; Dobson, R.J.B.; et al. Microduplications at the pseudoautosomal SHOX locus in autism spectrum disorders and related neurodevelopmental conditions. *J. Med. Genet.* **2016**, *53*, 536–47, doi:10.1136/jmedgenet-2015-103621.

32. Leblond, C.S.; Heinrich, J.; Delorme, R.; Proepper, C.; Betancur, C.; Huguet, G.; Konyukh, M.; Chaste, P.; Ey, E.; Rastam, M.; et al. Genetic and functional analyses of SHANK2 mutations suggest a multiple hit model of autism spectrum disorders. *PLoS Genet.* **2012**, *8*, e1002521, doi:10.1371/journal.pgen.1002521.

33. Cosemans, N.; Vandenhove, L.; Vogels, A.; Devriendt, K.; Van Esch, H.; Van Buggenhout, G.; Olivié, H.; de Ravel, T.; Ortibus, E.; Legius, E.; et al. The clinical relevance of intragenic NRXN1 deletions. *J. Med. Genet.* **2020**, *57*, 347–355, doi:10.1136/jmedgenet-2019-106448.

34. Upners, E.N.; Jensen, R.B.; Rajpert-De Meyts, E.; Dunø, M.; Aksglaede, L.; Juul, A. Short stature homeobox-containing gene duplications in 3.7% of girls with tall stature and normal karyotypes. *Acta Paediatr.* **2017**, *106*, 1651–1657, doi:10.1111/apa.13969.

35. Mahmuda, N. Al; Yokoyama, S.; Munesue, T.; Hayashi, K.; Yagi, K.; Tsuji, C.; Higashida, H. One Single Nucleotide Polymorphism of the TRPM2 Channel Gene Identified as a Risk Factor in Bipolar Disorder Associates with Autism Spectrum Disorder in a Japanese Population. *Dis. (Basel, Switzerland)* **2020**, *8*, doi:10.3390/diseases8010004.

36. Nardone, S.; Sams, D.S.; Reuveni, E.; Getselter, D.; Oron, O.; Karpuj, M.; Elliott, E. DNA methylation analysis of the autistic brain reveals multiple dysregulated biological pathways. *Transl. Psychiatry* **2014**, *4*, e433, doi:10.1038/tp.2014.70.

37. Tan, T.Y.; Sedmík, J.; Fitzgerald, M.P.; Halevy, R.S.; Keegan, L.P.; Helbig, I.; Basel-Salmon, L.; Cohen, L.; Straussberg, R.; Chung, W.K.; et al. Bi-allelic ADARB1 Variants Associated with Microcephaly, Intellectual Disability, and Seizures. *Am. J. Hum. Genet.* **2020**, *106*, 467–483, doi:10.1016/j.ajhg.2020.02.015.

38. Lin, A.; Hou, Q.; Jarzylo, L.; Amato, S.; Gilbert, J.; Shang, F.; Man, H.-Y. Nedd4-mediated AMPA receptor ubiquitination regulates receptor turnover and trafficking. *J. Neurochem.* **2011**, *119*, 27–39, doi:10.1111/j.1471-4159.2011.07221.x.

39. Broix, L.; Jagline, H.; Ivanova, E.; Schmucker, S.; Drouot, N.; Clayton-Smith, J.; Pagnamenta, A.T.; Metcalfe, K.A.; Isidor, B.; Louvier, U.W.; et al. Mutations in the HECT domain of NEDD4L lead to AKT-mTOR pathway deregulation and cause periventricular nodular heterotopia. *Nat. Genet.* **2016**, *48*, 1349–1358, doi:10.1038/ng.3676.

40. Manojlovic, Z.; Earwood, R.; Kato, A.; Stefanovic, B.; Kato, Y. RFX7 is required for the formation of cilia in the neural tube. *Mech. Dev.* **2014**, *132*, 28–37, doi:10.1016/j.mod.2014.02.001.

41. Ishizuka, K.; Kimura, H.; Wang, C.; Xing, J.; Kushima, I.; Arioka, Y.; Oya-Ito, T.; Uno, Y.; Okada, T.; Mori, D.; et al. Investigation of Rare Single-Nucleotide PCDH15 Variants in Schizophrenia and Autism Spectrum Disorders. *PLoS One* **2016**, *11*, e0153224, doi:10.1371/journal.pone.0153224.

42. Stone, J.L.; Merriman, B.; Cantor, R.M.; Geschwind, D.H.; Nelson, S.F. High density SNP association study of a major autism linkage region on chromosome 17. *Hum. Mol. Genet.* **2007**, *16*, 704–15, doi:10.1093/hmg/ddm015.

43. Veerappa, A.M.; Saldanha, M.; Padakannaya, P.; Ramachandra, N.B. Family-based genome-wide copy number scan identifies five new genes of dyslexia involved in dendritic spinal plasticity. *J. Hum. Genet.* **2013**, *58*, 539–47, doi:10.1038/jhg.2013.47.

44. Sanchez-Pulido, L.; Ponting, C.P. TMEM132: an ancient architecture of cohesin and immunoglobulin domains define a new family of neural adhesion molecules. *Bioinformatics* **2018**, *34*, 721–724, doi:10.1093/bioinformatics/btx689.

45. Hadley, D.; Wu, Z.-L.; Kao, C.; Kini, A.; Mohamed-Hadley, A.; Thomas, K.; Vazquez, L.; Qiu, H.; Mentch, F.; Pellegrino, R.; et al. The impact of the metabotropic glutamate receptor and other gene family interaction networks on autism. *Nat. Commun.* **2014**, *5*, 4074, doi:10.1038/ncomms5074.

46. Namjou, B.; Marsolo, K.; Caroll, R.J.; Denny, J.C.; Ritchie, M.D.; Verma, S.S.; Lingren, T.; Porollo, A.; Cobb, B.L.; Perry, C.; et al. Phenome-wide association study (PheWAS) in EMR-linked pediatric cohorts, genetically links PLCL1 to speech language development and IL5-IL13 to Eosinophilic Esophagitis. *Front. Genet.* **2014**, *5*, 401, doi:10.3389/fgene.2014.00401.

47. Baldan, F.; Gnan, C.; Franzoni, A.; Ferino, L.; Allegri, L.; Passon, N.; Damante, G. Genomic Deletion Involving the IMMP2L Gene in Two Cases of Autism Spectrum Disorder. *Cytogenet. Genome Res.* **2018**, *154*, 196–200, doi:10.1159/000489001.

48. Guo, H.; Peng, Y.; Hu, Z.; Li, Y.; Xun, G.; Ou, J.; Sun, L.; Xiong, Z.; Liu, Y.; Wang, T.; et al. Genome-wide copy number variation analysis in a Chinese autism spectrum disorder cohort. *Sci. Rep.* **2017**, *7*, 44155, doi:10.1038/srep44155.

49. Kushima, I.; Aleksic, B.; Nakatochi, M.; Shimamura, T.; Okada, T.; Uno, Y.; Morikawa, M.; Ishizuka, K.; Shiino, T.; Kimura, H.; et al. Comparative Analyses of Copy-Number Variation in Autism Spectrum Disorder and Schizophrenia Reveal Etiological Overlap and Biological Insights. *Cell Rep.* **2018**, *24*, 2838–2856, doi:10.1016/j.celrep.2018.08.022.

50. Ikeda, M.; Tomita, Y.; Mouri, A.; Koga, M.; Okochi, T.; Yoshimura, R.; Yamanouchi, Y.; Kinoshita, Y.; Hashimoto, R.; Williams, H.J.; et al. Identification of novel candidate genes for treatment response to risperidone and susceptibility for schizophrenia: integrated analysis among pharmacogenomics, mouse expression, and genetic case-control association approaches. *Biol. Psychiatry* **2010**, *67*, 263–9, doi:10.1016/j.biopsych.2009.08.030.
