# Supplementary material for: Evaluation of Chromosome Microarray Analysis in a Large Cohort of Females with Autism Spectrum Disorders: A Single Center Italian Study
Source: J Pers Med. 2020 Oct 9;10(4):160. doi: 10.3390/jpm10040160 (PMC7720139; doi:10.3390/jpm10040160)
Supplement: Supplementary file 1 [file jpm-10-00160-s001.zip › Supplementary Table S1_rev_fms - def.docx]

| **Table S1 – Phenotipic characteristics of participants.** For each participant are reported the age at last examination (mo = months; y = years), IQ level, ADOS module, level of ASD symptoms and ADOS scores, language development at the last examination (IQ level: N >70; D ≤ 70; SA: Social Affect domain scores; RRB: Restricted and Repetitive Behaviors domain scores; CSS: Calibrated Severity Score; Language: V = verbal, NV = non-verbal). | | | | | | | | |
| --- | --- | --- | --- | --- | --- | --- | --- | --- |
| **Individuals with positive CMA** | | | | | | | | |
| **ID** | **Age at last examination** | **IQ level** | **ADOS**  **Module** | **SA** | **RRB** | **CSS** | **Level of ASD symptoms** | **Language** |
| P1 | 7y/9mo | D | - | - | - | - | - | V |
| P2 | 4y/5mo | D | 1 | 8 | 10 | 10 | severe | NV |
| P3 | 3y/6mo | D | - | - | - | - | moderate | NV |
| P4 | 5y/10mo | N | 2 | 7 | 7 | 6 | moderate | V |
| P5 | 3y/11mo | D | - | - | - | - | - | NV |
| P6 | 3y/8mo | N | 1 | 9 | 8 | 9 | severe | V |
| P7 | 21 mo | N | Toddler | 6 | 1 | 5 | mild | V |
| P8 | 3y/10mo | D | - | - | - | - | - | NV |
| P9 | 3y/5mo | N | 3 | 7 | 7 | 7 | moderate | V |
| P10 | 3y/10mo | D | 1 | 10 | 6 | 8 | severe | NV |
| P11 | 3y/1mo | D | 1 | 7 | 6 | 5 | moderate | V |
| P12 | 3y/2mo | D | - | - | - | - | moderate | V |
| P13 | 6y/4mo | N | 2 | 7 | 6 | 7 | moderate | V |
| P14 | 14y | N | 3 | 5 | 5 | 4 | mild | V |
| P15 | 3y/7mo | N | 1 | 7 | 9 | 6 | moderate | V |
| P16 | 6y/4mo | N | - | - | - | - | moderate | V |
| P17 | 5y/1mo | D | 1 | 8 | 8 | 8 | severe | NV |
| P18 | 13y | N | 3 | 8 | 5 | 7 | moderate | V |
| P19 | 7y/3mo | D | 1 | 7 | 6 | 6 | moderate | V |
| P20 | 11y | N | 3 | 3 | 5 | 3 | mild | V |
| P21 | 5y/2mo | D | - | - | - | - | moderate | V |
| P22 | 3y | D | - | - | - | - | - | NV |
| P23 | 3y/11m | N | 2 | 5 | 1 | 3 | mild | V |
| P24 | 3y/9mo | N | 2 | 3 | 6 | 3 | mild | V |
| P25 | 3y/4mo | N | 1 | 3 | 1 | 2 | below the ASD cut-off | V |
| P26 | 3y/1mo | na | 1 | 9 | 5 | 6 | moderate | NV |
| P27 | 4y/5mo | D | - | - | - | - | - | NV |
| P28 | 2y/7mo | D | 1 | 9 | 10 | 10 | severe | NV |
| P29 | 4y/5mo | D | - | - | - | - | - | NV |
| **Individuals with negative CMA** | | | | | | | | |
| P30 | 3y/7mo | N | - | - | - | - | below the ASD cut-off | V |
| P31 | 13y | N | 3 | 5 | 7 | 5 | moderate | V |
| P32 | 2y/10mo | D | Toddler | 10 | 8 | 10 | severe | V |
| P33 | 6y/1mo | N | 2 | 3 | 7 | 4 | mild | V |
| P34 | 10y | D | 2 | 5 | 6 | 6 | moderate | V |
| P35 | 4y/11mo | N | 1 | 10 | 10 | 10 | severe | V |
| P36 | 2y/4mo | N | Toddler | 5 | 5 | 5 | mild | V |
| P37 | 2y/11mo | N | 1 | 8 | 6 | 7 | moderate | V |
| P38 | 4y/1mo | D | - | - | - | - | moderate | NV |
| P39 | 4y/3mo | N | 1 | 3 | 8 | 3 | mild | V |
| P40 | 17y | N | - | - | - | - | - | V |
| P41 | 4y | D | 1 | 6 | 10 | 8 | severe | V |
| P42 | 4y/4mo | D | 1 | 9 | 10 | 10 | severe | NV |
| P43 | 2y/2mo | N | Toddler | 4 | 7 | 3 | mild | V |
| P44 | 7y/3mo | N | 2 | 9 | 7 | 8 | severe | V |
| P45 | 3y/9mo | N | Toddler | 9 | 10 | 10 | severe | V |
| P46 | 8y | D | 2 | 10 | 6 | 9 | severe | V |
| P47 | 8y | D | - | - | - | - | - | V |
| P48 | 3y/4mo | N | Toddler | 7 | 6 | 7 | severe | V |
| P49 | 5y/5mo | D | 1 | 9 | 10 | 10 | severe | NV |
| P50 | 2y/11mo | N | 1 | 6 | 1 | 5 | moderate | V |
| P51 | 23 mo | D | - | - | - | - | severe | NV |
| P52 | 4y/4mo | N | 2 | 10 | 10 | 10 | severe | V |
| P53 | 12y | D | - | - | - | - | - | NV |
| P54 | 4y/3mo | D | 1 | 2 | 10 | 4 | mild | NV |
| P55 | 2y/8mo | D | 1 | 6 | 9 | 7 | moderate | NV |
| P56 | 3y/5mo | N | 1 | 5 | 7 | 7 | moderate | NV |
| P57 | 5y/9mo | na | 1 | 10 | 8 | 9 | severe | V |
| P58 | 3y/1mo | N | 1 | 5 | 8 | 6 | moderate | NV |
| P59 | 23mo | N | 1 | 5 | 8 | 6 | moderate | V |
| P60 | 3 y/1 mo | N | 1 | 8 | 5 | 5 | moderate | V |
| P61 | 2 y/10 mo | D | 1 | 10 | 7 | 7 | moderate | V |
| P62 | 4 y/1 mo | D | - | - | - | - | - | V |
| P63 | 5 y/2 mo | N | 2 | 7 | 6 | 6 | moderate | V |
| P64 | 3 y | D | - | - | - | - | moderate | NV |
| P65 | 2 y/5 mo | D | 1 | 9 | 10 | 7 | moderate | V |
| P66 | 9 y | N | 3 | 9 | 7 | 9 | severe | V |
| P67 | 2 y/8 mo | D | 1 | 6 | 9 | 6 | moderate | V |
| P68 | 3 y/9 mo | N | 1 | 4 | 9 | 4 | mild | V |
| P69 | 6 y/7 mo | D | 1 | 7 | 5 | 6 | moderate | V |
| P70 | 2 y/9 mo | D | 1 | 8 | 8 | 8 | severe | V |
| P71 | 8 y/11 mo | D | - | - | - | - | - | NV |
| P72 | 3 y/9 mo | N | 2 | 9 | 6 | 8 | severe | V |
| P73 | 4 y/1 mo | N | - | - | - | - | - | V |
| P74 | 3 y/4 mo | N | 1 | 2 | 6 | 1 | below the ASD cut-off | V |
| P75 | 3 y | N | 1 | 8 | 10 | 10 | severe | V |
| P76 | 3 y/7 mo | N | 1 | 4 | 8 | 4 | mild | V |
| P77 | 5 y/7 mo | N | - | - | - | - | severe | V |
| P78 | 2 y/8 mo | D | 1 | 9 | 9 | 7 | severe | V |
| P79 | 3 y/2 mo | N | - | - | - | - | mild | V |
| P80 | 2 y/1 mo | N | Toddler | 10 | 7 | 10 | severe | NV |
| P81 | 3 y/1 mo | D | 1 | 8 | 10 | 7 | moderate | V |
| P82 | 4 y/1 mo | D | 1 | 9 | 10 | 10 | severe | NV |
| P83 | 2 y/11 mo | na | 1 | 7 | 7 | 6 | moderate | NV |
| P84 | 17 y | D | 4 | 2 | 10 | 4 | mild | V |
| P85 | 2 y/8 m | D | 1 | 7 | 6 | 7 | moderate | V |
| P86 | 2 y/9 m | D | 1 | 6 | 9 | 5 | moderate | V |
| P87 | 2 y/9 m | D | 1 | 8 | 10 | 10 | severe | NV |
| P88 | 3 y/1 mo | N | 1 | 6 | 7 | 6 | moderate | V |
| P89 | 3 y | N | - | - | - | - | mild | NV |
| P90 | 7 y/3 mo | N | 1 | 3 | 7 | 3 | mild | V |
